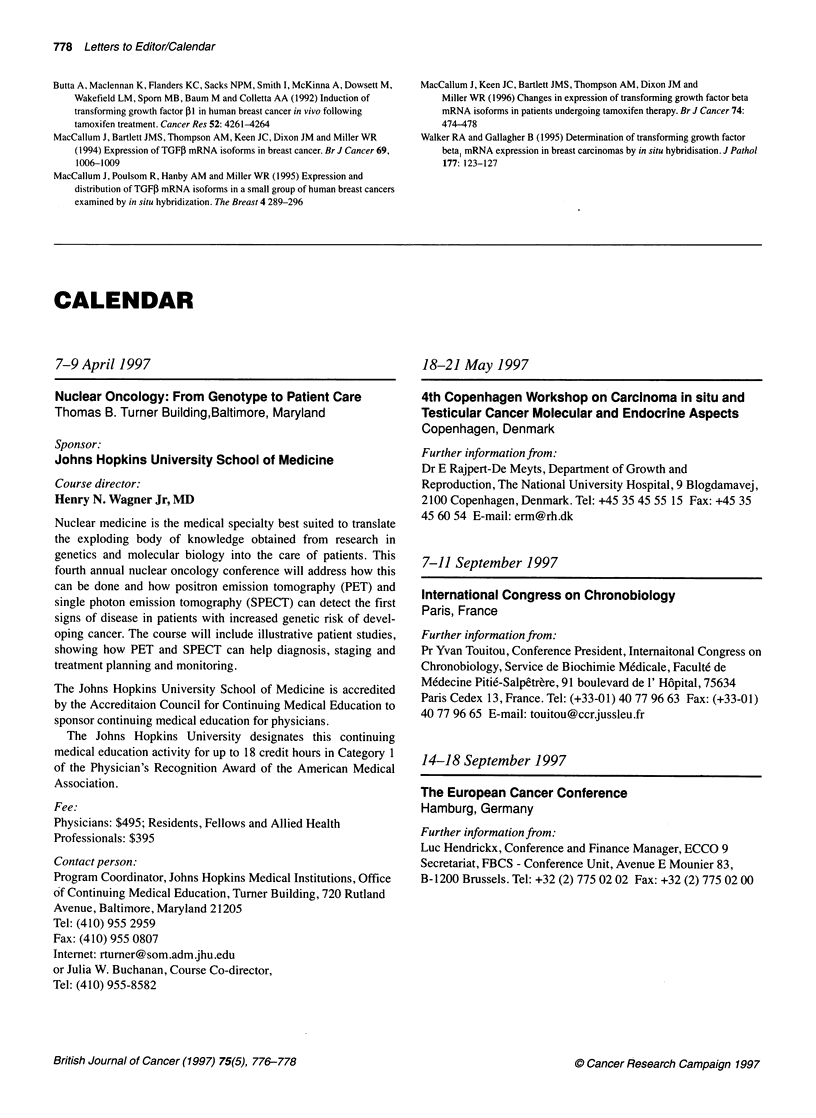# Calendar

**Published:** 1997

**Authors:** 


### CALENDAR

### *7–9 April 1997*

**Nuclear Oncology: From Genotype to Patient Care**
Thomas B. Turner Building,Baltimore, Maryland

*Sponsor:*
**Johns Hopkins University School of Medicine**

*Course director:*
**Henry N. Wagner Jr, MD**

Nuclear medicine is the medical specialty best suited to translate the exploding body of knowledge obtained from research in genetics and molecular biology into the care of patients. This fourth annual nuclear oncology conference will address how this can be done and how positron emission tomography (PET) and single photon emission tomography (SPECT) can detect the first signs of disease in patients with increased genetic risk of developing cancer. The course will include illustrative patient studies, showing how PET and SPECT can help diagnosis, staging and treatment planning and monitoring.

The Johns Hopkins University School of Medicine is accredited by the Accreditaion Council for Continuing Medical Education to sponsor continuing medical education for physicians.

The Johns Hopkins University designates this continuing medical education activity for up to 18 credit hours in Category 1 of the Physician's Recognition Award of the American Medical Association.

*Fee:*
Physicians: $495; Residents, Fellows and Allied Health Professionals: $395

*Contact person:*
Program Coordinator, Johns Hopkins Medical Institutions, Office of Continuing Medical Education, Turner Building, 720 Rutland Avenue, Baltimore, Maryland 21205
Tel: (410) 955 2959
Fax: (410) 955 0807
Internet: rturner@som.adm.jhu.edu
or Julia W. Buchanan, Course Co-director,
Tel: (410) 955-8582

### *18–21 May 1997*

**4th Copenhagen Workshop on Carcinoma in situ and Testicular Cancer Molecular and Endocrine Aspects**
Copenhagen, Denmark

*Further information from:*
Dr E Rajpert-De Meyts, Department of Growth and Reproduction, The National University Hospital, 9 Blogdamavej, 2100 Copenhagen, Denmark. Tel: +45 35 45 55 15 Fax: +45 35 45 60 54 E-mail: erm@rh.dk

### *7–11 September 1997*

**International Congress on Chronobiology**
Paris, France

*Further information from:*
Pr Yvan Touitou, Conference President, Internaitonal Congress on Chronobiology, Service de Biochimie Médicale, Faculté de Médecine Pitié-Salpêtrère, 91 boulevard de l' Hôpital, 75634 Paris Cedex 13, France. Tel: (+33-01) 40 77 96 63 Fax: (+33-01) 40 77 96 65 E-mail: touitou@ccr.jussleu.fr

### *14–18 September 1997*

**The European Cancer Conference**
Hamburg, Germany

*Further information from:*
Luc Hendrickx, Conference and Finance Manager, ECCO 9 Secretariat, FBCS - Conference Unit, Avenue E Mounier 83, B-1200 Brussels. Tel: +32 (2) 775 02 02 Fax: +32 (2) 775 02 00

© Cancer Research Campaign 1997